# Vascular dementia and the cholinergic pathways

**DOI:** 10.1590/S1980-57642008DN10100002

**Published:** 2007

**Authors:** Eliasz Engelhardt, Denise Madeira Moreira, Jerson Laks

**Affiliations:** 1 MD, PhD, Cognitive and Behavioral Neurology Unit-INDC/UFRJ.; 2 MD, PhD, Neuroradiology Unit-INDC/UFRJ, Radiology Unit of PróCardíaco-RJ Hospital.; 3 MD, PhD, Coordinator of CDA-IPUB/UFRJ.

**Keywords:** anatomy, vascular dementia, cognitive impairment, cholinergic fibers, anatomia, demência vascular, comprometimento cognitivo, fibras colinérgicas

## Abstract

Vascular cognitive impairment/vascular dementia have been the subject of a large number of studies, due to their high prevalence and broad preventive and compensatory therapeutic potential. The knowledge of the cerebral anatomy correlated to the vascular territories of irrigation enables understanding of clinical manifestations, as well as classification into the several types of syndromic presentations. The central cholinergic system exercises important neuromodulatory functions on cerebral circuits related to cognitive and behavioral integration, as well as on vasomotor control related to cerebral blood flow adjustments. The acquisition of data on the anatomy of the cholinergic pathways, including the localization of the nuclei of the basal prosencephalon and the routes of their projections, established an important milestone. The knowledge of the vascular distribution and of the trajectories of the cholinergic pathways allows identification of the strategic points where a vascular lesion can cause interruption. The ensuing denervation leads to cholinergic hypofunction in the involved territories. This information proves important to better evaluate the sites of vascular lesions, emphasizing their strategic localizations in relation to the cholinergic pathways, and offering more robust foundations for treatment aiming at enhancing cholinergic activity.

## Abstract (pt)

Comprometimento cognitivo vascular/demência vascular vem sendo objeto de numerosos estudos, levando em conta sua alta prevalência e as amplas possibilidades terapêuticas preventivas e compensatórias. O conhecimento da anatomia cerebral correlacionado ao dos territórios vasculares de irrigação permite a compreensão das manifestações clínicas, assim como a classificação dos diversos tipos de apresentações sindrômicas. O sistema colinérgico central exerce funções neuromoduladoras importantes dos circuitos relacionados à integração cognitiva e comportamental, além do controle vasomotor relacionado aos ajustes do fluxo sanguíneo cerebral. A obtenção de dados sobre a anatomia das vias colinérgicas, incluindo a localização dos núcleos do prosencéfalo basal e os trajetos das suas projeções, estabeleceu um marco importante. O conhecimento da distribuição vascular e do percurso das vias colinérgicas permite identificar pontos estratégicos onde a lesão vascular pode causar sua interrupção. A desnervação que se segue causa hipofunção colinérgica dos territórios acometidos. Essas informações são importantes para melhor avaliar os locais das lesões vasculares, enfatizando suas localizações estratégicas em relação às vias colinérgicas, oferecendo, desse modo, bases mais sólidas para o tratamento que visa aumentar a atividade colinérgica.

The study of cholinergic hypofunction in Alzheimer’s disease (AD) is already more then two decades old, and has recently been extended to other dementing illnesses, such as the Lewy body diseases (dementia with Lewy bodies, dementia and Parkinson’s disease) and vascular dementia (VaD). This knowledge underpins the widely known cholinomimetic treatment strategy, with the efficacious use of cholinesterase inhibitors^1-4^. Degeneration of the cholinergic nuclei of the basal prosencephalon (BP) and the derangement of their projections making up the cholinergic pathways can be seen in several primary dementing diseases.

Lesions of the BP and/or of the cholinergic pathways can be found at varied points of their course in VaD. The same can be seen in mixed presentations, the most commonly described being AD+CVD and MD (AD+VaD)^5^.

The knowledge of the cholinergic system, both in normal and pathological states, is important to fully understand how the cholinergic treatment strategy works in VaD and what benefits it offers.

## The cholinergic nuclei of the basal prosencephalon

The central cholinergic system is made up of several clusters of neurons distributed across different levels of the brain. The BP lies in the basal part and comprises four clusters or groups of cholinergic neurons, the large nucleus basalis of Meynert (nbM) being among them. The others include the medial nucleus of the septum (nmS) and the nuclei of the diagonal band of Broca, along with the vertical (ndbBvl) and the horizontal (ndbBhl) limbs^6-7^.

The groups of cholinergic neurons in these nuclei are named according to the Ch nomenclature, and are found in nmS (Ch1), in ndbBvl (Ch2), in ndbBhl (Ch3), and in nbM (Ch4)^6,8^.

The nmS plus ndbBvl comprise about 20 000 neurons, with 3 200 cholinergic neurons, in each hemisphere^9-10^. The nbM has about 200 000 neurons in each hemisphere, subdivided into sectors related with particular cortical areas, in approximately a mediolateral and anteroposterior topography^11-13,6,14-15^.

All cholinergic neurons express acetylcholinesterase (AChE) and choline acetyltransferase (ChAT). The Ch1- Ch4 clusters differ by the presence of neurons (about 90%) containing the nerve growth factor receptor (NGFr), tirosine kinase (TRKa) and the neurotrophine receptor (p75NTR), not found in cholinergic neurons at other levels^6^.

## The cholinergic system and its functions

The central cholinergic system exercises important functions including neuromodulation of brain circuits related to cognitive and behavioral integration^16-20^ and to vasomotor control.

Vasomotor control is related to modulation of brain blood flow, exerted through two mechanisms:

- circumscribed enhancement of perfusion related to increased neural activity in a given area caused by cholinergic stimulation, corresponding to ‘functional hyperemia’ resulting from neurovascular metabolic coupling^21^ and
- vasodilator action on arteries of varied caliber, mainly on terminal ramifications (arterioles, capillaries) accomplished through muscarinic receptors localized close to astrocytic terminations (gliovascular complexes) with liberation of nitric oxide to the smooth muscular fibers and pericytes^22-25^.

Vasomotor control has been studied in animal models, where vasodilatation was shown by cholinergic stimulation^22,26-29^. An increase of perfusion was also seen in normal subjects and patients with AD or VaD with PET and SPECT imaging related to cholinergic intervention (use of cholinesterase inhibitors)^5,30-34^.

Thus, this double activity, tissular and vascular, makes the cholinergic system important in normal functional condition. On the other hand, its hypofunction becomes an important target for interventions aiming to enhance its modulatory activity.

## The anatomy of the cholinergic pathways

The projections from the BP cholinergic groups are directed toward several subcortical and cortical brain regions^6,8-10,12,35-39^.

The projections to the hippocampal formation and entorhinal cortex originate mainly from Ch1-Ch2 and have a route that accompanies the fornix. The terminals reach mainly the CA2-CA4 sectors of the hippocampus and the dentate gyrus, with a lesser density to sector CA1 and subiculum.

The Ch3 group is directed to olfactory areas, reached through the medial prosencephalic fascicle.

The projections to other regions of the cortex originate in the Ch4 group and constitute two bundles, the medial and the lateral. Fibers detach from these bundles and supply subcortical regions and cerebral cortex.

The *medial cholinergic pathway* originates from the nbM, passes through the white matter of the straight and medial orbital gyri, around the rostrum of the corpus callosum and accompanies the cingulum bundle until the splenium, where it continues to the retrosplenial white matter. This pathway supplies ramifications to the medial orbitofrontal, subcallosal, cingulate, pericingulate, and retrosplenial cortical regions.

The *lateral cholinergic pathway* arises from the nbM and forms a compact bundle that subdivides in the capsular and perisylvian divisions that run through the external capsule and the claustrum, ramify widely in the centrum semiovale and subcortical white matter, and distribute fibers to the inferior frontal, frontoparietal operculum, temporal, insular, and para-hippocampal neocortex. The amygdala also receives fibers from the lateral pathway.

**Figure 1:**
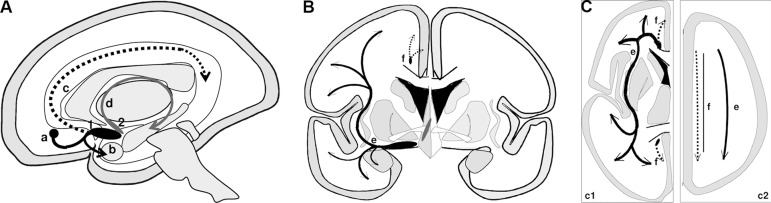
Basal prosencephalon and projections.

**Figure 2:**
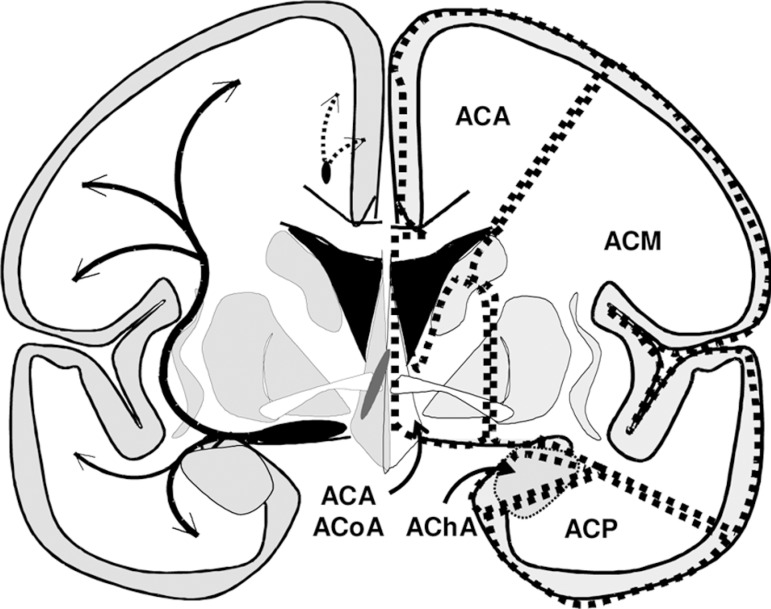
Coronal schema of the brain – the cholinergic pathways (left side) (medial path=black-interrupted; lateral path=black-continuous) and the limits of the main vascular territories (right side).

The cortical layers of all cytoarchitectonic regions present a dense cholinergic innervation. The density of the cholinergic axons is higher in the more superficial cortical layers (I, II, and superior parts of layer III). There is a significant difference in the global density of the cholinergic axons among the several cytoarchitetonic regions. The highest fiber density is observed in the central limbic structures, such as the hippocampal formation and amygdala, followed by the cortical paralimbic areas, entorhinal and cingulate cortex; the cholinergic innervation of the unimodal and heteromodal associative areas is of intermediary density, while that of the primary sensory areas is the lowest^6,12,18^ (Table 1, Figures 1 and 2). The cortical cholinergic axons are mainly amyelinic and establish symmetric and asymmetric synapses with a large number of cortical and subcortical neurons. It is likely that part of the released ACh and the action it exerts is extra-synaptic, reaching neurons and neuroglia relatively distant from the site of neurotransmitter release by diffusion (volume transmission)^40-43^.

**Table 1:** Brain cholinergic system - cholinergic groups, main projections and most important destinations of the basal prosencephalon.

Basal prosencephalon - nuclei	Bundles/fascicles		Destination
Medial nucleus of septum (Ch1)	nmS	fornix	hippocampal formation
nucleus of diagnonal band of Broca -	ndbBvl		entorhinal cortex
vertical limb (Ch2)			retrosplenial cortex
		medial prosencephalic	hypothalamus
Nucleus of the diagonal band of Broca -	ndbBhl	medial prosencephalic	olfactory bulb
horizontal limb (Ch3)			
basal nucleus of Meynert (Ch4)	nbM	ansa peduncularis	amygdala
		(ventral amigdalofugal)	
		medial pathway	*alo- and mesocortex*
			medial orbitofrontal, subcallosal, cingulate,
			pericingulate, retrosplenial
		lateral pathway	*neocortex*
			inferior frontal, dorsal frontoparietal, frontoparietal
			opercular, temporal (superior, middle and inferior),
			insular, inferotemporal, para-hippocampal

## The cholinergic pathways and the cerebral vascular territories

The cholinergic projections of the septo-hippocampal path present a fairly compact constitution, running through the fornix to reach the hippocampal formation. The Meynert-cortical projections present a relatively compact origin, but once outside the basal ganglia territory, at the level of the centrum semiovale, the lateral pathway presents a fanlike distribution to reach their destination areas, while the medial pathway runs mainly through the cingulum and distributes ramifications along its route^38^.

The main cerebral arteries – anterior cerebral artery (ACA), middle cerebral artery (MCA), anterior communicating artery (ACoA), posterior communicating artery (PCoA), anterior choroidal artery (AChA) – provide irrigation of the territories where the cholinergic projections travel^5,46-51^ (Table 2, Figure 2).

**Table 2:** Territories of the cerebral arteries related to cholinergic structures or their routes.

Artery	Territory
ACA	BP (Ch3 and Ch4-pt), septal region, frontal (basal), subcallosal area, cingulum, centrum semiovale (pt)
MCA	BP (Ch4-pt), claustrum, external and extreme capsules, centrum semiovale (pt)
PCA	centrum semiovale (pt)
AChA	BP (pt)
ACoA	BP (Ch1 e Ch2), septum, subcallosal area, cingulum (anterior pt), fornix (columns)
PCoA	----

ACA, anterior cerebral artery; MCA, middle cerebral artery; PCA, posterior cerebral artery; AChA, anterior choroidal artery; ACoA, anterior communicating artery; PCoA, posterior communicating artery; BP, basal prosencephalon; pt, part.

## The cholinergic pathways and cerebrovascular disease

**Figure 3:**
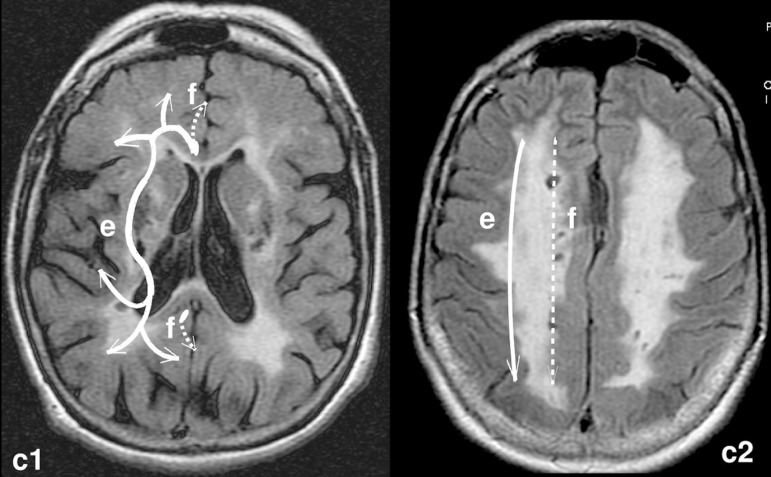
RM-FLAIR. Axial sections of the brain of a case with extensive subcortical demyelination. The cholinergic pathways are depicted to the left side (medial path= white -interrupted, lateral path=white continuous) on two levels (similar to Figure 1C).

Ischemic or hemorrhagic processes represent the several cerebrovascular pathologies that can cause tissue damage and interruption of the cholinergic pathways. The ischemic processes cause territorial infarcts, watershed infarcts, lacunes, white matter demyelination, affecting areas of varied size^52-56^. It is possible to localize the points where lesions can interrupt these pathways by considering the routes of the cholinergic pathways^38^ and the vascular territories (Table 2, Figure 2, Figure 3).

The lesions of the BP, severely affecting the septal area and/or the nbM, can occur due to ischemia in the territories of the ACA, ACoA and MCA. The projection of the septal area to the hippocampal formation, via the fornix, can be interrupted by lesions in the territory of the ACoA. The interruption of the wide Meynert-cortical projections may stem from a variety of lesion sites. The main medial pathway can be interrupted at any point of its route in the cingulum due to pathology in the ACA and ACoA territories, while the ramifications of this pathway, with a more radiating distribution, can be injured in territories of the same arteries at a variety of points. The main lateral pathway can be affected in its sublenticular and paralenticular route (external capsule and claustrum) due to lesions in the territories of the ACA, ACoA and MCA, and its wide and fanlike course can be affected in the white matter of the centrum semiovale, irrigated mainly by the ACA, MCA and PCA (Figure 2C, Figure 3).

Two neuropathologic studies indicate a relationship between CVD and the interruption of these pathways, besides the anatomic relations between the cholinergic pathways and the vascular territories.

One of these studies was conducted using brain tissue from a patient with CADASIL (cerebral autosomic dominant arteriopathy with subcortical infarcts and leucoencepalopathy), a disease that can be considered a model for pure CVD (VaD). The material was stained with a histochemical technique to show AChE revealing cholinergic denervation in several cortical areas, except for the hippocampal formation and entorhinal cortex. Even in the more affected areas a number of AChE positive fibers were seen. The cholinergic neurons of the nbM were undamaged, as verified by techniques for NGFr and AChE^57^.

The other study was performed using brain tissue of patients with VaD of the Binswanger subtype. The material was stained with histochemical and imunnohistochemical techniques to show AChE and ChAT. This material revealed severe reduction of AChE and ChAT positive fibers in the external capsule and claustrum, in comparison to controls. The nbM had large neurons preserved, but showed some chromatolytic changes and numerical reduction.

A neuroimage-neuropathological correlation was possible for some of the patients. MRI showed hyperintensities in the frontal periventricular white matter, extending to the subinsular white matter (where the external capsule is found). The brains of the same patients at autopsy showed loss of myelin in the corresponding regions^58^. Therefore, underpinned by these two paradigmatic studies, we can state that CVD may cause interruption of segments of the cholinergic pathways, leading to denervation and consequent cholinergic hypofunction of the affected territories.

Cholinergic hypofunction, variable according to the lesioned segment of the cholinergic pathways, causes integrative dysfunction of the target brain structures and disturbances of vasomotor control with consequent reduction in brain blood flow of the affected areas^5,59^. These functional data gave rise to the proposal of a ‘cholinergic neurovascular hypothesis^22^.

Recently, two studies were dedicated to the relationship between the cholinergic pathways and the white matter hyperintensities, correlated to the clinical manifestations of VCI/VaD, with the aim of staging scales. These proposals relate the white matter lesions with their localization in relation to the cholinergic pathways. The staging was graduated according to the visually evaluated extension, and number of lesions localized, along the anatomical known routes of the cholinergic pathways. One of these rating scales classified the lesions in the cholinergic pathways as minimal (absence of lesions in nbM and absence of hyperintensities in medial pathway or external capsule), moderate (lesions in external capsule plus in lateral pathway) and severe (nbM infarction or external capsule plus lateral pathway hyperintensities or large hyperintensities in lateral pathway or hyperintensities in both lateral and medial pathways)^60^. The other, more detailed rating, proposes an evaluation on 4 slices (low external capsule, high external capsule, corona radiata and centrum semiovale), separated into 10 regions. The severity of white matter lesions was visually rated on a 3-point scale (0-3) for each region, and weighted (1-4) to account for the decreasing concentration of cholinergic fibers as they project and fan out in the white matter^61^. The results of these studies suggest that the localization of the hyperintensities in the white matter holds special importance, considering that some of these may occur at strategic points and may be related to measurable clinical manifestations^60,61^.

Thus, the knowledge of the anatomy of the cholinergic pathways and their relation to those vascular territories where an interruption can occur, allied to the consequent clinical manifestations, enable better evaluation of CVD clinical expression. It may also be able to lend a more solid basis for treatment strategies, such as the cholinergic approach.

## Conclusion

CVD can cause clinical symptoms defining VCI/VaD according to its extension and localization. Two mechanisms play a role: one corresponding to tissue lesions of cortical areas and subcortical regions, including white matter responsible for disconnection related manifestations, while the other is related to the interruption of the cholinergic pathways at various localizations along their routes, producing manifestations consequent to cholinergic denervation which result in a hypocholinergic state of the affected territories.

Knowledge of cognitive-behavioral and vasomotor functions of the cholinergic system, allied to that of the anatomical localization of the course of its pathways, is important to better assess the sites of vascular lesion. Such knowledge permits strategic points of the cholinergic pathways to be highlighted and provides more solid bases for use of cholinergic therapeutic strategies.
